# The mChoice App, an mHealth Tool for the Monitoring of Preexposure Prophylaxis Adherence and Sexual Behaviors in Young Men Who Have Sex With Men: Usability Evaluation

**DOI:** 10.2196/59780

**Published:** 2025-02-28

**Authors:** Fabiana Cristina Dos Santos, Maeve Brin, Mary R Tanner, Carla A Galindo, Rebecca Schnall

**Affiliations:** 1 School of Nursing, Columbia University, 560 W 168th St, New York, NY, 10032, United States, 1 212-305-5756; 2 Division of HIV Prevention, Centers for Disease Control and Prevention, Atlanta, GA, United States

**Keywords:** HIV prevention, data visualization, patient-reported health information, mHealth, digital health, usability, human immunodeficiency virus, preexposure prophylaxis, men who have sex with men, apps, HIV, PrEP

## Abstract

**Background:**

Mobile health (mHealth) apps provide easy and quick access for end users to monitor their health-related activities. Features such as medication reminders help end users adhere to their medication schedules and automatically record these actions, thereby helping manage their overall health. Due to insufficient mHealth tools tailored for HIV preventive care in young men who have sex with men (MSM), our study evaluated the usability of the mChoice app, a tool designed to enhance preexposure prophylaxis (PrEP) adherence and promote sexual health (eg, encouraging the use of condoms and being aware of the partner’s HIV status and PrEP use).

**Objective:**

This study aimed to apply systematic usability evaluations to test the mChoice app and to refine the visualizations to better capture and display patient-reported health information.

**Methods:**

Usability testing involved heuristic evaluations conducted with 5 experts in informatics and user testing with 20 young MSM who were taking or were eligible to take PrEP.

**Results:**

End users demonstrated satisfaction with the appearance of the mChoice app, reporting that the app has an intuitive interface to track PrEP adherence. However, participants highlighted areas needing improvement, including chart titles and the inclusion of “undo” and “edit” buttons to improve user control when recording PrEP use.

**Conclusions:**

Usability evaluations involving heuristic experts and end users provided valuable insights into the mChoice app’s design. Areas for improvement were identified, such as enhancing chart readability and providing additional user controls. These findings will guide iterative refinements, ensuring that future versions of the app better address the needs of its target audience and effectively support HIV prevention.

## Introduction

The incidence of HIV, although having declined modestly overall in the United States, remains a significant public health problem for men who have sex with men (MSM) [1,2]. Notably, young MSM aged 13 to 34 years represent 58% of the estimated HIV infections in 2021, indicating a pressing need for targeted intervention in this group [2]. Preexposure prophylaxis (PrEP) is a highly effective biomedical prevention strategy to reduce HIV incidence and curb the HIV epidemic [3-10]. When taken as prescribed (daily or at least 4 times per week), PrEP reduces HIV transmission by up to 99% among MSM [7-9,11-13]. While awareness of PrEP has increased among MSM [14,15], those who are most disproportionately affected have been found to be less aware of PrEP [15], and overall, widespread uptake among those with the greatest indications for PrEP remains low [14-17].

To overcome this clinical and public health challenge, technological interventions, such as mobile health (mHealth) apps, have emerged to support public health care initiatives [18]. However, despite the potential of mHealth for delivering sexual health and HIV prevention awareness, mHealth is underutilized for supporting PrEP adherence [19,20]. In response, our study team developed mChoice, an innovative PrEP adherence monitoring app, which combines the CleverCap smart pill bottle with WiseApp, allowing end users to self-monitor and manage their medication adherence. The CleverCap pill bottle dispenses the prescribed medication and interfaces with WiseApp for real-time tracking. The app was created by Compliance Meds Technology and has been used in prior studies among persons living with HIV [10,21-25]. It was adapted for the mChoice study. The app’s key functionalities include monitoring PrEP adherence, visualizing data trends, and documenting sexual behavior, particularly among young MSM from diverse backgrounds.

A notable issue in the proliferation of mHealth apps is the development of this technology with minimal input from end users, leading to poor design, inadequate consideration of user needs, and ultimately, poor usability [26]. Poorly designed apps, lacking in usability, are prone to misuse, underutilization, and failure to achieve their intended objectives.

To ensure the best utilization of mHealth apps, it is essential to understand their usability, keeping in mind target end users (eg, young MSM), tasks (eg, PrEP adherence management and sexual health tracking), and cultural contexts (eg, language and beliefs). Adherence to PrEP is crucial for its effectiveness in preventing HIV, yet many young MSM face challenges such as inconsistent medication schedules and limited access to tailored support tools. The mChoice app was designed to address these barriers by providing intuitive features, including medication reminders, adherence tracking, and functions to support sexual health decision-making. In this study, we sought to apply systematic usability evaluations to test the mChoice app and to refine the functions to better capture and display patient-reported health information.

## Methods

### Ethical Considerations

The Columbia University Institutional Review Board reviewed and approved all study activities (approval number AAAT8812). Participants signed informed consent forms prior to participating in usability testing. In our dataset, each participant was assigned an identifier, and information was deidentified at the point of analysis.

### Heuristic Evaluation

#### Sample

Usability testing of the mChoice app included heuristic evaluation conducted with experts in informatics and end-user testing conducted with young MSM. Five experts were recruited as evaluators in accordance with Nielsen’s recommendation to include 3 to 5 experts in usability testing [27]. Of the 5 experts, 4 had a PhD in Nursing with expertise in human-computer interaction, interface design, and usability testing. The experience of the experts varied between 7 and 23 years, with several impactful publications in the field of informatics.

#### Procedure

**Figure 1.**
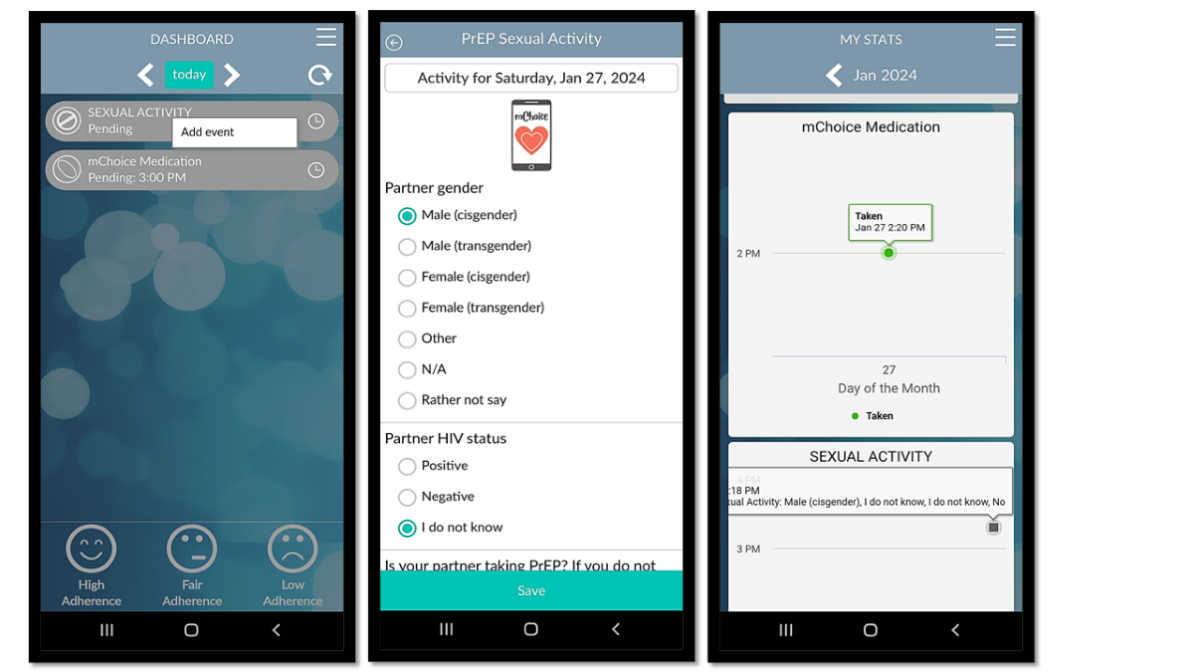
Sexual activity log. A confidential and secure feature for users to record their sexual activities, such as gender of partner, whether a condom was used, and the HIV status of their partner. Users of the on-demand/intermittent (2-1-1) preexposure prophylaxis (PrEP) regimen can use this feature to trigger subsequent PrEP dose reminders following sexual activity (ie, one pill 24 hours and one pill 48 hours after sexual activity).

The experts were provided with the mChoice app and asked to complete a session in the app through case scenarios that represented the main functions of the system and think-aloud methodology [28]. The mChoice app required a password, and all study data were encrypted and stored on secure HIPAA-compliant servers at Columbia University. The experts were asked to describe what they were thinking, seeing, and doing as they completed the following 10 scenarios: (1) log in to the CleverCap app; (2) complete a sexual activity log; (3) mark your PrEP dose for today as “Taken”; (4) view tomorrow’s pending PrEP dose; (5) edit your sexual activity log; (6) delete your sexual activity log; (7) view information on PrEP dosing over the past month; (8) send a chat to the study team; (9) watch a video about an HIV story; and (10) find PrEP information (see Figures1  2 illustrating app functions and Figure 3 demonstrating the CleverCap smart pill bottle). Following their use of the app, the experts completed an online Heuristic Evaluation Checklist to assess how the system adhered to Nielsen’s 10 usability principles [27] using Qualtrics XM, an online survey software. Each question ranged from 0 (not a usability problem) to 4 (usability catastrophe). A member of the research team analyzed the experts’ comments regarding usability problems to identify areas of usability concern that could be targeted for improvement. The mean usability problem severity scores were calculated for each of the 10 usability heuristics.

**Figure 2.**
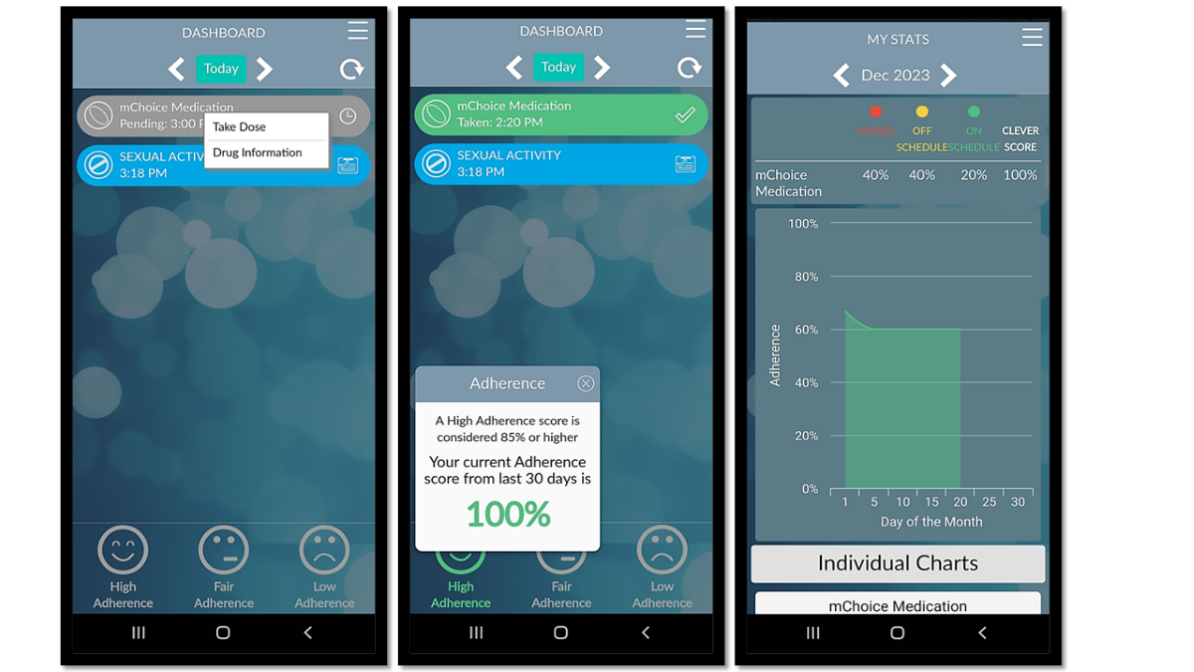
Preexposure prophylaxis (PrEP) adherence tracking. A simple interface for users to track PrEP intake, facilitating adherence monitoring over time. This feature is linked with the CleverCap device, a smart pill bottle that tracks when users take their medication. PrEP tracking is also customizable for users on different PrEP regimens (ie, 2-1-1, injectable, or daily oral dose) to ensure the timely intake of PrEP medication. For instance, users on injectable PrEP will be alerted of their upcoming appointment or need for their PrEP injection.

**Figure 3.**
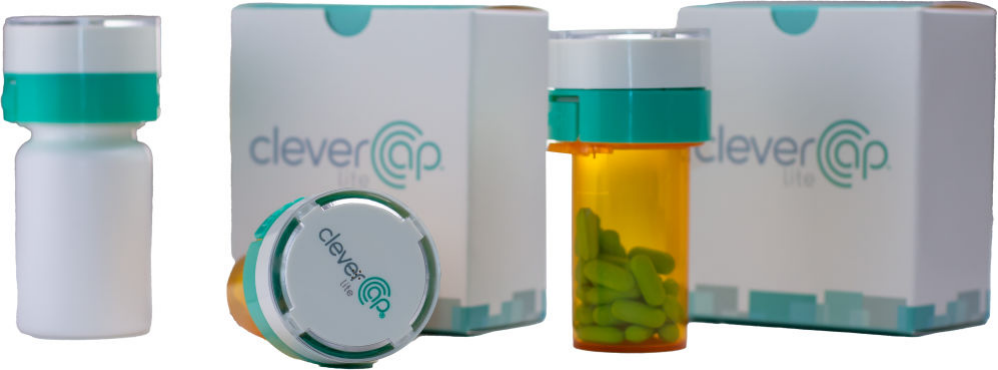
CleverCap device. A smart pill bottle linked with the mChoice app to track preexposure prophylaxis (PrEP) medication.

### End-User Testing

#### Sample

Twenty end users were recruited for the usability testing. The eligibility criteria were young MSM between 18 and 39 years old, English speaking, living in New York or Alabama, and taking or eligible to take PrEP. The inclusion criteria were designed to ensure the mChoice app was evaluated by its intended target audience, ensuring a diverse usability evaluation. Potential end users were contacted using a research database and invited to participate in the study.

#### Procedure

During usability testing, the end users were provided with a brief explanation of the mChoice app and its functions. The end users were directed to complete the same 10 scenarios completed by the experts in the heuristic evaluation. Sessions took place remotely via Zoom meetings (Zoom Communications, Inc), and a member of the team was present during sessions to provide guidance when an end user was unable to move through a task independently and to take notes. Following their use of the app, the end users completed a survey using the Qualtrics XM software.

The survey included demographics and two validated measures of usability. The first usability measure was the self-reported ease of use measured by using the Health Information Technology Usability Evaluation Scale (Health-ITUES) [29,30]. This 20-item tool is designed to support customization at the item level to match the health information technology while retaining standardization at the construct level. It has been demonstrated to be useful for evaluating the usability of mHealth [31]. It is scored on a 5-point Likert scale ranging from 5 (strongly agree) to 1 (strongly disagree), where higher scores indicate a system that is easier to use. The second measure was the short version of the Post-Study System Usability Questionnaire (PSSUQ), a 16-item survey that assesses users’ perceived satisfaction with a system [32]. Scoring is based on a 7-point Likert scale ranging from 1 (strongly agree) to 7 (strongly disagree). Lower scores indicate satisfaction with the system. Through validated measures, the end users contributed feedback on the app’s functionality and user experience.

Data analysis was performed using R statistical software (version 4.1.2; R Foundation for Statistical Computing) to analyze the usability measures. The mean scores were calculated for each survey.

## Results

### Heuristic Evaluation

Overall, the severity scores ranged from 0.8 to 2.2 for the 10 items on the heuristic checklist, where scores closest to 0 indicate a more usable system. The mean severity scores for each heuristic item are presented in Table 1. The area identified as the most in need of improvement was “user control and freedom,” where the experts identified that the app did not have an “undo” button or “edit” button for the PrEP medication entry.

The next most identified area for improvement was the “visibility of the system status,” for which experts identified the lack of one “add” button to add a new sexual event. To improve the “visibility of the system status,” the experts suggested we provide one “edit” button for modifying recorded sexual encounters and incorporate a separate “add” button for logging new encounters, especially in instances of multiple events. Additionally, “consistency and standards” was an area of concern with difficult terminology in charts. “Error prevention” was another concern, and experts highlighted the importance of one “edit” button to edit medication time as an issue that might impede system usability. Experts provided favorable feedback on the app’s user experience. One expert (Expert 2) reported, “the app is very simple and intuitive interface…and it’s nice that the button changes color with pleasant colors.”

The rating score ranged from 0 (ie, best) to 4 (ie, worst), with a score of 0 indicating no usability problem, 1 indicating a cosmetic problem only, 2 indicating a minor usability problem, 3 indicating a major usability problem, and 4 indicating a usability catastrophe.

**Table 1.** Mean severity scores for items on the heuristic evaluation.

Heuristic principles	Mean (SD) severity score	Experts’ comments to identify the areas of usability concerns
User control and freedom	2.2 (1.3)	“No undo or edit for the medication entry. This is a big problem.” (Expert 5)
Visibility of the system status	1.6 (1.1)	“…When adding a sexual encounter record, it is not clear whether it edits or adds a new one, so there should be two buttons, one for edit and one to add a new one. The screen when you use edit also shows the old entry, which implies you are editing it, not starting a new one, but actually it is starting a new one. That is confusing.” (Expert 3)
Consistency and standards	1.6 (1.1)	“Not clear if the clever score is just adherence or if it means something else.” (Expert 4) “…I think in the menu it says stats. Rather than stats, it’d be adherence stats or adherence tracking over time. You know something that makes it really clear what people are clicking on.” (Expert 2)
Error prevention	1.6 (1.1)	“When entering the time of a medication, it might be easy to miss the AM/PM change. I don’t think there’s any design change to be made for this, but rather the user needs to be able to edit their entry if they forget to adjust this.” (Expert 4)
Match between the system and the real world	1.4 (1.3)	“Would like to see one section specific to PrEP information.” (Expert 5)
Recognition rather than recall	1.4 (0.9)	“Some of the main features can be moved to more obvious places.” (Expert 1) “Organization for videos could be improved, especially if you plan to add more content.” (Expert 4)
Help and documentation	1.4 (1.3)	“Information buttons and an introduction to the app would be helpful.” (Expert 1)
Help users recognize, diagnose, and recover from errors	1.2 (1.1)	“When the dose was missed, no way to edit if it was a mistype.” (Expert 5)
Aesthetic and minimalist design	1.2 (1.1)	“Graphs need clearer tiles and more labels.” (Expert 1) “Need more medication information or guidance if adherence is low.” (Expert 5)
Flexibility and efficiency of use	0.8 (1.1)	“…Search function for videos. Tags for topics would be good, too.” (Expert 4)

### End-User Testing

The end-user group for evaluating the mChoice app’s usability consisted entirely of men, with the majority identifying as homosexual (14/20, 70%). The mean (SD) age was 28 (3.4) years. Half of the end users were White, and 75% (15/20) were non-Hispanic or Latino. Considering education, 50% (10/20) had a college degree. Two specific measures were employed for the usability assessment: the Health-ITUES and the PSSUQ, as detailed in Table 2.

The Health-ITUES scale, where a higher score indicates better usability, showed that “perceived ease of use” scored the highest with a mean (SD) score of 4.6 (0.5), suggesting a favorable user assessment. However, “user control” received the lowest score in this category, with a mean (SD) score of 3.8 (0.9), corroborating the heuristic evaluator’s ratings.

Conversely, the PSSUQ, where a lower score indicates better usability, reflected different aspects of the mChoice performance. “System quality” scored the lowest with a mean (SD) of 1.7 (1.2), while “interface quality” had the highest score with a mean (SD) score of 3.0 (1.8). These results suggested a positive user experience with notable ease of use and system quality strengths. However, there were areas for improvement, particularly in enhancing user control and interface quality.

**Table 2.** Usability measures.

Measures	Mean (SD) score
Health-ITUES^a^	
Perceived ease of use	4.6 (0.5)
Impact	4.1 (0.8)
Perceived usefulness	4.0 (0.8)
User control	3.8 (0.9)
Overall Health-ITUES score	4.2 (0.4)
PSSUQ^b^	
System quality	1.7 (1.2)
Information quality	2.4 (1.3)
Interface quality	3.0 (1.8)
Overall PSSUQ score	2.3 (0.6)

a Health-ITUES: Health Information Technology Usability Evaluation Scale (rating the score from 5 being the best score to 1 being the worst score).

b PSSUQ: Post-Study System Usability Questionnaire (rating the score from 1 being the best score to 7 being the worst score).

## Discussion

### Principal Results

Our study evaluated the mChoice app’s usability in enhancing PrEP adherence for young MSM. Overall, heuristic experts and end users demonstrated satisfaction with the mChoice app, reporting that it is a simple and intuitive interface for tracking PrEP adherence. Graphs, charts, and icons are the main features of mHealth apps that help end users track their goals, habits, and achievements [33,34]. The mChoice app combines iconography (eg, emoticons) and color-coded charts to provide clear and immediate feedback on PrEP adherence. These diverse visualizations allow end users to interpret their data in several formats. The app includes emoticons in different colors (green for high adherence, red for missed doses, and yellow for off-schedule doses) to represent adherence status. For instance, a sad emoticon in red for low adherence might highlight medication adherence challenges and prompt self-reflection on areas needing improvement. Such immediate visual feedback can motivate end users to improve their medication routine. In addition, graphs showed the trend of PrEP medication adherence over time, including evidence-based information on the percentage of missed, off-schedule, and on-time doses.

While prior research has indicated that varied data visualization options help match different end users’ preferences [33] and color-coded visual cues can aid in quick decision-making and interpretation of health data [35-38], our study did not directly assess end-user interpretations of these visual elements. The mChoice app incorporates these evidence-based design principles, but further research would be needed to evaluate their impact on user comprehension and medication adherence behaviors.

Our findings suggest that the visual elements available in the mChoice app offer a positive end-user experience and function accessibility that could assist in timely medication intake and monitor adherence. However, our heuristic evaluation highlighted areas needing improvement, particularly in the need for clearer chart titles and more labels to exemplify each component (eg, Clever score), underscoring the importance of refinement of the mChoice design for optimized end-user experiences.

The accuracy of the information collected through the mChoice app was another area of attention that heuristic experts raised concerns, particularly highlighting the absence of “undo,” “edit,” and “add” buttons. These limitations restricted user control, potentially affecting the accuracy of patients’ health information input. For instance, the absence of “undo” and “edit” buttons could lead to inaccuracies if end users mistakenly enter the time of their PrEP dose intake. Additionally, the lack of an “add” button for recording multiple sexual events was seen as an important barrier, restricting end users from documenting their sexual event entries accurately. These findings underscore the need for enhanced app capabilities to ensure accurate and comprehensive user input and customizations. Similarly, other studies [39,40] reported concerns about user experiences and data accuracy in mobile apps, reinforcing the findings of our study.

The actionable finding from this study is to reevaluate the functions of the mChoice app after integrating end-user feedback and addressing heuristic violations. Iterative usability evaluations should be considered as a best practice in the app development process to ensure the effectiveness of the app’s future iterations.

### Limitations

A limitation of our study was the convenience sample, recruited from a database that included participants who had participated in past research and expressed interest in future studies. Additionally, while our sample targeted young MSM, English-speaking men, and those living in New York or Alabama, this demographic representation might limit our ability to understand how the app might perform for medication adherence across diverse populations. Given that PrEP users come from varied cultural, linguistic, and socioeconomic backgrounds, our findings may not be generalizable to groups whose cultural beliefs, language preferences, and health care engagement patterns differ from those of our study population. While end users were central for evaluating the usability of the mChoice app through validated quantitative measures, qualitative data were collected only from experts during heuristic evaluations. In this study, we did not evaluate the artificial intelligence or algorithmic components that could enhance personalization and medication adherence predictions. Despite these limitations, we identified areas of improvement to refine the mChoice app. In addition, this study enriched the body of research on evaluating the mHealth usability for HIV prevention. This was achieved by employing end users and heuristic experts to assess this innovative app designed for PrEP adherence and HIV prevention.

### Conclusions

Our usability study of the mChoice app involved rigorous evaluations through interactive heuristic evaluations and end-user testing. The results demonstrated the app’s simplicity and user-friendly interface, showcasing its potential in monitoring health-related activities such as PrEP adherence. Further research could explore how these usability enhancements might influence user engagement and behavior change to support HIV prevention efforts.

## Abbreviations

Health-ITUES: Health Information Technology Usability Evaluation Scale
mHealth: mobile health
MSM: men who have sex with men
PrEP: preexposure prophylaxis
PSSUQ: Post-Study System Usability Questionnaire
